# Pediatric Autoimmune Hepatitis in a Patient Who Presented With Erythema Nodosum: A Case Report

**Published:** 2012-01-20

**Authors:** Zohreh Kavehmanesh, Fatemeh Beiraghdar, Amin Saburi, Ali Hajihashemi, Susan Amirsalari, Morteza Movahed

**Affiliations:** 1 Research Center for Gastroenterology and Liver Diseases, Baqiyatallah University of Medical Sciences, Tehran, IR Iran; 2 Nephrology and Urology Research Center, Baqiyatallah University of Medical Sciences, Tehran, IR Iran; 3 Health Research Center, Baqiyatallah University of Medical Sciences, Tehran, IR Iran; 4 Chemical Injury Research Center, Baqiyatallah University of Medical Sciences, Tehran, IR Iran; 5 Department of Pediatric, Baqiyatallah University of Medical Sciences, Tehran, IR Iran; 6 Department of Pathology, Baqiyatallah University of Medical Sciences, Tehran, IR Iran

**Keywords:** Pediatrics, Hepatitis, Autoimmune, Erythema Nodosum

## Abstract

**Background:**

Autoimmune hepatitis (AIH) is a form of chronic hepatitis with unclear causative factors and is characterized by immunological and auto-immunological manifestations. Several extrahepatic manifestations, such as other autoimmune disorders, are associated with AIH. AIH with dermatological conditions as the initial manifestation is rare. We report the case of AIH in which erythema nodosum (EN) was the first manifestation.

**Case Presentation:**

An 8-year-old girl with several persistent dermatological lesions was referred to our hospital several months ago. Her skin had nodular, painful, dry, and erythematous lesions, predominantly on the extensor areas of both the legs, with some erythematous patches on her face. Physical examination revealed that she had hepatosplenomegaly as well. Skin biopsy indicated EN. The results of the laboratory tests showed increased levels of several liver enzymes. The patient's International Autoimmune Hepatitis Group (IAIHG) score was a definite indicator of AIH. The results of liver biopsy indicated AIH. Other causes of EN and abnormal liver function were ruled out. The only obvious cause of skin lesions was chronic inflammation due to an autoimmune response. The patient was treated for AIH, and her skin lesions along with other signs and symptoms resolved.

**Conclusions:**

AIH can present with protean clinical manifestations, and is thus associated with the risk of delayed diagnosis. Dermatological manifestations, including EN, could indicate a serious disease, and further investigation might be required. AIH should be considered as the possible diagnosis in such cases.

## 1. Background

Autoimmune hepatitis (AIH), previously known as chronic active hepatitis or lupoid hepatitis, is a form of chronic hepatitis of uncertain or unknown etiology characterized by immunological and autoimmunological manifestations and is usually accompanied by the presence of circulating autoantibodies and high serum globulin concentrations [1]. AIH occurs in adults and children of all ages, mainly affecting females, and it occasionally has a fluctuating course, with periods of increased or diminished activity. AIH responds dramatically to immunosuppressive therapy, but when left untreated, it progressively and rapidly develops into cirrhosis and liver failure [2][3]. AIH is responsible for approximately 2-5% of chronic liver diseases in children. Its incidence in Europe is 0.69 per 100,000 of the adult population. The prevalence of pediatric AIH has not been documented [2][3][4][5]. AIH often follows a more acute and aggressive course in children and young adults than in middle-aged and elderly patients [6].

The recent classification of AIH is based on the type of serum autoantibodies that are produced, although there is little evidence supporting the role of these antibodies in the pathogenesis of this disorder. Two main types of AIH have been delineated. Type 1 (Classic) AIH occurs mostly in females of all age groups; it is characterized by the presence of anti-nuclear antibody (ANA) and/or anti-smooth muscle antibody (SMA). Type 2 AIH commonly occurs in girls and young women and is characterized by the presence of anti-liver kidney microsomal antibody type 1 (anti-LKM-1) [1][3][6]. The cause of AIH is not clearly known, but overactivity and dysregulation of immune cells (especially CD8 lymphocytes) is thought to be the main cause of this disease [1]. The signs and symptoms of AIH vary and are similar to those of other autoimmune disorders; AIH patients may present with nonspecific symptoms, such as extrahepatic manifestations. One of the organs involved in AIH is skin. Pruritus, urticaria pigmentosa, vitiligo, erythema annulare centrifugum, and cutaneous polyarteritis nodosa are some of the skin manifestations of AIH [7][8][9][10]. Erythema nodosum (EN) is a skin lesion associated with some autoimmune diseases such as inflammatory bowel syndrome and Behçet syndrome, and some non-autoimmune disorders such as tuberculosis, bacterial or deep fungal infection, sarcoidosis, and cancer [11][12][13]. EN seems to occur less frequently in children than in adults [14]. Although AIH is an autoimmune disease and EN is not unusual in autoimmune disorder, there are few reports of an association between EN and AIH [15]. We discuss the case of pediatric AIH in which EN was the initial presentation.

## 2. Case Presentation

**Figure 1:**
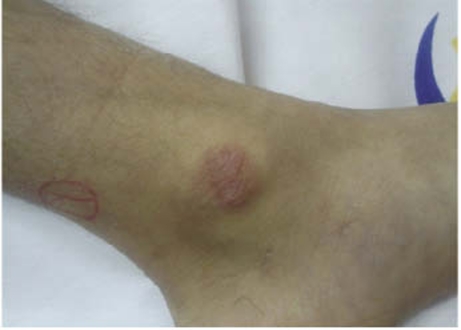
The Skin Lesions Were Present on Both the Legs and Were Tender,Erythematous, and Predominantly Nodular in Shape, Without Any Discharge (Before Treatment)

An 8-year-old girl was under observation at our dermatology outpatient clinic because of several skin lesions that had persisted for 5-6 months. Initially, the patient was diagnosed as having eczema and topical treatment was started. Over a 2-month period, the patient showed no improvement and showed the additional clinical presentations of epigastric abdominal discomfort, weakness, asthenia, occasional nausea, and vomiting and was referred to the pediatric ward. The skin lesions were present on both the legs and were tender, erythematous, nodular, with a predominantly nodular shape and without any discharge (Figure 1). Skin biopsy of the erythematous nodule revealed full thickness subcutaneous fat infiltration with lymphocytic white blood cell (panniculitis) without any evidence of vasculitis. The mentioned findings were consistent with a diagnosis of erythema nodosum [16]. She did not have a history of musculoskeletal complaint, pharyngitis, blood transfusion, or any medications, all of which was consistent with her condition. She had no family history of chronic liver disease or any autoimmune disorders either. Physical examination revealed mild hepatosplenomegaly, without any other peripheral stigmata of liver disease. Her vital signs were stable.

The results of the laboratory tests were as follows: WBC count, 4,500 × 10(6) / lit (neutrophils: 39%, lymphocytes: 55%, and others: 6%); hypochromic microcytic anemia (hemoglobin: 10.3 g/dL, MCV: 71); platelets, 174,000/μL; erythrocyte sedimentation rate, 24; creatinine level, 0.9 mg/dL; total bilirubin, 1.6 mg/dL and direct bilirubin, 0.4 mg/dL; serum aspartate aminotransfarase (AST) level, 348 IU/L (normal level, up to 40 IU/L); alanine aminotransferase (ALT) level, 555 IU/L (normal level, up to 40 IU/L), alkaline phosphatase (ALP) level, 395 IU/L (normal level, up to 1800 IU/L in pediatric patients); and lactate dehydrogenase (LDH) level, 612 IU/L (normal level, up to 450 IU/L). The results of hemostatic screening tests (for the prothrombin time and international normalized ratio) were normal. Serum protein electrophoresis showed total protein level of 6.9 g/dL (normal range, 6.0-8.4 g/dL), albumin level of 3.6 g/dL (normal range, 3.5-5.0 g/dL), and globulin level of 2.97 g/dL (normal range, 2.3-3.5 g/dL). Her blood samples were not positive for HBs antigen, anti-HBc antibody, anti-hepatitis A virus immunoglobulin M, and anti-hepatitis C virus antibody. The results of the serological tests were negative for cytomegalovirus, Epstein-Barr virus, Toxoplasma, and Brucella. The streptococcal anti-streptolysin O titer was not remarkable. Smears of gastric secretion and the microbial culture were negative for the tuberculin bacilli as were the results of the Koch bacillus (BK) and purified protein derivative (PPD) skin tests. The results of the serological tests for anti-staphylococcal and anti-streptococcal antibodies were also negative. The serum and urine levels of ferritin, ceruloplasmin, α1-antitrypsin, and γ-glutamyl transpeptidase were normal. Urine analysis did not show any abnormalities, and there were no clinical and laboratory findings suggesting diabetes mellitus, thyroiditis, Graves' disease, or proliferative glomerulonephritis.

Abdominal ultrasonography showed increased liver size, with a coarse echotexture, but it did not show any evidence of portal hypertension and ascites. The results of chest computed tomography were unremarkable. Ophthalmological examination did not show any Kayser-Fleischer ring. An assessment of the immunoserological markers showed that anti-SMA was present at a titer of 1: 160, but serum ANA, anti-mitochondrial antibodies (AMA), and anti-LKM antibodies were not. The results of human leukocyte antigen typing were positive for DR3 and DR4. Other possible causes of EN were ruled out [12]. Liver biopsy revealed lymphocytic infiltration with a mild degree of fibrosis, rosette formation, and other histopathological findings (interface hepatitis), indicating chronic autoimmune hepatitis.

**Figure 2:**
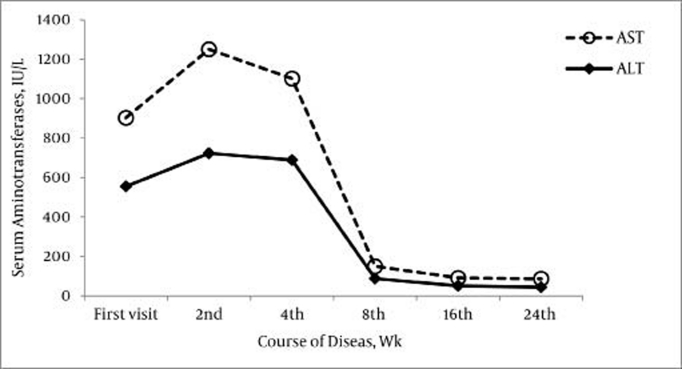
Changes in the Levels of Serum Aminotransferases During the Disease Course

The only obvious cause of skin lesions was chronic inflammation due to an autoimmune response. The patient was diagnosed as having type-1 AIH associated with EN, and a course of prednisolone with azathioprine was started in the second week. The patient scores for the diagnosis of AIH were calculated according to the International Autoimmune Hepatitis Group (IAIHG) revised scoring system [17]. The pre-treatment IAIHG score of the patient was 19, which is a definite indicator of AIH. During the first follow-up (after 4 weeks of therapy), abdominal discomfort, weakness, asthenia, and fatigue were entirely resolved, and the skin nodules were significantly improved. Serum aminotransferase levels were near normal. The changes in the levels of serum aminotransferases during treatment have been shown in Figure 2. The skin lesions completely remitted after 3 months of treatment. We started the treatment with the prednisolone (2 mg/ kg/ d) and azathioprine (2 mg/kg/ d). The immunosuppressive therapy was tapered with the administration of prednisone (0.5 mg/kg/day) and azathioprine (1 mg/kg/d) as a maintenance regimen when we found that the levels of serum aminotransferases remained normal the first follow up. Our patient is currently doing well, as indicated by the normal results of the laboratory tests and after the 6-month follow-up post-treatment.

## 3. Discussion

EN is a type of panniculitis (inflammation of subcutaneous adipose tissue in the skin) that is clinically characterized by acute-onset skin eruptions such as tender erythematous nodules located mostly over the extensor surfaces of the legs (especially shins) [16]. EN occurs in about 1-5 individuals per 100,000 persons. Various wide-spectrum general and local conditions can trigger EN. Streptococcal infections are the most common identifiable cause of EN, especially in children. Other bacterial and viral infections, drugs, sarcoidosis, inflammatory bowel disease, and autoimmune disorders are the other common causes of EN [12][14]. Sometimes, EN is the sign of an autoimmune disease such as ankylosing spondylitis, antiphospholipid antibody syndrome, Behçet's syndrome, Reiter's syndrome, rheumatoid arthritis, Sjögren's syndrome, systemic lupus erythematosus, Takayasu's arteritis, and Wegener's granulomatosis, which can be potentially relieved with immunosuppressive and supportive therapy [16][18]. EN is considered to be a reactive process that is caused by an inordinate response of the immune system to various trigger factors. The histopathological mechanism that explains the involvement of the immune system in EN is deposition of immune complexes and infiltration of neutrophils in the connective tissue septa of subcutaneous fat [16].

AIH, like other autoimmune diseases, is characterized by necroinflammation of the liver due to lymphocytic infiltrations, presence of circulating non-organ-specific autoantibodies, and hypergammaglobulinemia with an unknown cause. The causative factor of AIH is not clearly known; however, an extrinsic factor is believed to activate a T-cell-mediated response against hepatocyte antigen in patients with genetic susceptibility [1][2]. We report the case of an 8-year-old girl with AIH who presented with EN as the initial manifestation. AIH, like the other autoimmune disorders, involves various organs, especially, the connective tissues. However, skin involvement as the initial manifestation of AIH is rare and improbable [15]. Irregularities in the responses from the humoral and cellular immune systems in the case of both AIH and EN may explain the association between them. Our patient responded to treatment adequately and her cutaneous lesions resolved, which confirms the association between AIH and EN. AIH associated with EN is a rare clinical condition, where EN is the initial clinical manifestation. Sometimes, the initial clue to a hepatic disorder is a skin manifestation [10]. It is necessary for physicians to be well-versed with cutaneous presentations in liver disease, in order to diagnose the underlying disorder as soon as possible.

## 4. Conclusion

AIH can present with many misleading clinical manifestations, increasing the risk of delayed diagnosis. This case showed that in the case of obscure symptoms, a thorough investigation is required for accurate diagnosis of AIH. In the case of a child with elevated levels of liver enzymes, AIH should be considered as a possible diagnosis when the results of other laboratory tests are inconclusive. Dermatological disorders can be a presentation of serious diseases such as AIH, and further investigation may be required.

## References

[1] Krawitt EL (2006). Autoimmune hepatitis. N Engl J Med.

[2] Mieli-Vergani G, Heller S, Jara P, Vergani D, Chang MH, Fujisawa T, González-Peralta RP, Kelly D, Mohan N, Shah U, Murray KF (2009). Autoimmune hepatitis. J Pediatr Gastroenterol Nutr.

[3] Gregorio GV, Portmann B, Mowat AP, Vergani D, Mieli-Vergani G (1997). A 12-year-old girl with antimitochondrial antibody-positive autoimmune hepatitis. J Hepatol.

[4] Hodges JR, Millward-Sadler GH, Wright R (1982). Chronic active hepatitis: the spectrum of disease. Lancet.

[5] Yachha SK, Srivastava A, Chetri K, Saraswat VA, Krishnani N (2001). Autoimmune liver disease in children. J Gastroenterol Hepatol.

[6] Mieli-Vergani G, Vergani D (2009). Autoimmune hepatitis in children: what is different from adult AIH?. Semin Liver Dis.

[7] Rashtak S, Pittelkow MR (2008). Skin involvement in systemic autoimmune diseases. Curr Dir Autoimmun.

[8] Gulati S, Mathur P, Saini D, Mannan R, Kalra V (2004). Erythema annulare centrifugum with autoimmune hepatitis. Indian J Pediatr.

[9] Lee WJ, Kim CH, Chang SE, Lee MW, Choi JH, Moon KC, Koh JK (2009). A case of cutaneous polyarteritis nodosa in autoimmune hepatitis. Acta Derm Venereol.

[10] Satapathy SK, Bernstein D (2011). Dermatologic disorders and the liver. Clin Liver Dis.

[11] Albrecht J, Atzeni F, Baldini C, Bombardieri S, Dalakas MC, Demirkesen C, Yazici H, Mat C, Werth VP, Sarzi-Puttini P (2006). Skin involvement and outcome measures in systemic autoimmune diseases. Clin Exp Rheumatol.

[12] Schwartz RA, Nervi SJ (2007). Erythema nodosum: a sign of systemic disease. Am Fam Physician.

[13] Tantisirin O, Puavilai S (2003). Long-term follow-up of erythema nodosum. J Med Assoc Thai.

[14] Labbe L, Perel Y, Maleville J, Taieb A (1996). Erythema nodosum in children: a study of 27 patients. Pediatr Dermatol.

[15] Cervia M, Parodi A, Rebora A (1982). Chronic active hepatitis and erythema nodosum. Arch Dermatol.

[16] Requena L, Sanchez Yus E (2007). Erythema nodosum. Semin Cutan Med Surg.

[17] Ebbeson RL, Schreiber RA (2004). Diagnosing autoimmune hepatitis in children: is the International Autoimmune Hepatitis Group scoring system useful?. Clin Gastroenterol Hepatol.

[18] Weinstein M, Turner D, Avitzur Y (2005). Erythema nodosum as a presentation of inflammatory bowel disease. CMAJ.

